# A novel prognostic signature of immune‐related genes for patients with colorectal cancer

**DOI:** 10.1111/jcmm.15443

**Published:** 2020-06-21

**Authors:** Jun Wang, Shaojun Yu, Guofeng Chen, Muxing Kang, Xiaoli Jin, Yi Huang, Lele Lin, Dan Wu, Lie Wang, Jian Chen

**Affiliations:** ^1^ Department of Surgery the Second Affiliated Hospital Zhejiang University School of Medicine Hangzhou China; ^2^ Department of Surgical Oncology the Second Affiliated Hospital Zhejiang University School of Medicine Hangzhou China; ^3^ Bone Marrow Transplantation Center of the First Affiliated Hospital Institute of Immunology Zhejiang University School of Medicine Hangzhou China

**Keywords:** bioinformatics, colorectal cancer, immunogenomic landscape, prognostic signature, TCGA

## Abstract

Colorectal cancer (CRC) is one of the most commonly diagnosed cancers with an estimated 1.8 million new cases worldwide and associated with high mortality rates of 881 000 CRC‐related deaths in 2018. Screening programs and new therapies have only marginally improved the survival of CRC patients. Immune‐related genes (IRGs) have attracted attention in recent years as therapeutic targets. The aim of this study was to identify an immune‐related prognostic signature for CRC. To this end, we combined gene expression and clinical data from the CRC data sets of The Cancer Genome Atlas (TCGA) into an integrated immune landscape profile. We identified a total of 476 IRGs that were differentially expressed in CRC vs normal tissues, of which 18 were survival related according to univariate Cox analysis. Stepwise multivariate Cox proportional hazards analysis established an immune‐related prognostic signature consisting of *SLC10A2*, *FGF2*, *CCL28*, *NDRG1*, *ESM1*, *UCN*, *UTS2* and *TRDC*. The predictive ability of this signature for 3‐ and 5‐year overall survival was determined using receiver operating characteristics (ROC), and the respective areas under the curve (AUC) were 79.2% and 76.6%. The signature showed moderate predictive accuracy in the validation and GSE38832 data sets as well. Furthermore, the 8‐IRG signature correlated significantly with tumour stage, invasion, lymph node metastasis and distant metastasis by univariate Cox analysis, and was established an independent prognostic factor by multivariate Cox regression analysis for CRC. Gene set enrichment analysis (GSEA) revealed a relationship between the IRG prognostic signature and various biological pathways. Focal adhesions and ECM‐receptor interactions were positively correlated with the risk scores, while cytosolic DNA sensing and metabolism‐related pathways were negatively correlated. Finally, the bioinformatics results were validated by real‐time RT‑qPCR. In conclusion, we identified and validated a novel, immune‐related prognostic signature for patients with CRC, and this signature reflects the dysregulated tumour immune microenvironment and has a potential for better CRC patient management.

## 1 INTRODUCTION

Colorectal cancer (CRC) is the third most commonly diagnosed cancer worldwide and ranks second in terms of cancer‐related mortality.^1^ An estimated 1.8 million new cases and 881 000 CRC‐related deaths were recorded in 2018 alone.^1^ Recent screening programs have reported a decrease in both the incidence and mortality of CRC,^2^ and new therapies have doubled the overall survival (OS) of patients with advanced CRC.^3^ However, the OS of CRC patients is still low as they are usually diagnosed at the advanced‐stage.^4^, ^5^ Therefore, it is essential to identify novel therapeutic, prognostic and diagnostic biomarkers for CRC.

Immunotherapy is now part of the standard treatment regimen for many solid tumours,^6^ as the immune system is frequently dysregulated in cancer patients and inextricably linked with tumour growth.^7^ Anti‐cancer immunotherapeutic approaches are classified into six categories: oncolytic virus therapy, adjuvant immunotherapy and cytokines, adoptive cell therapy, therapeutic vaccines, checkpoint inhibitors and monoclonal antibodies.^8^ However, insufficient immune response has long been a concern, especially for checkpoint inhibitors targeting the programmed death 1 (PD‐1) and PD‐1 ligands (PD‐L1s) in CRC.^9^ Especially, use of immune checkpoint inhibitors (ICIs) has little or no clinical activity in most metastatic CRC patients.^10^ The response to ICI therapy depends on several key factors, including mutation load (high levels of tumour neoantigen), tumour‐infiltrating lymphocytes and regulatory checkpoint receptors.^11^ As seen in CRC patients with microsatellite stability (MSS), the main obstacle for ICI is low tumour immunogenicity^12^, ^13^ because of the presence of fewer neoantigens,^14^ resulting in fewer infiltrating CD8 + T cells and fewer strongly positive for PD‐1.^15^ This makes CRC become one of the tumours in which immunotherapy has been shown less effective, in relation to different classes. Therefore, it is essential to characterize the immune‐related genes (IRGs) in CRC to optimize treatment.

Gene microarrays and RNA‐sequencing have identified several prognostic biomarkers for human cancers in recent years. For instance, cytokines,^16^ heat shock protein beta 3,^17^ cyclin D1,^18^ clusterin^19^ and RBP7^20^ are established prognostic markers for CRC. In addition, non‐coding RNAs such as microRNAs,^21^, ^22^ long non‐coding RNAs^23^, ^24^ and circRNAs^25^ are increasingly reported to be associated with the survival of cancer patients. Several immune‐related prognostic signatures have also been established for multiple cancer types.^26^ For example, an IRG prognostic signature consisting of *SPAG11A, PTH2R, IL17C, IL11, FAM3B, CTGF* and *AGTR1* was constructed based on TCGA data for papillary thyroid cancer.^27^ Wang et al^28^ analysed the gene expression profiles of TCGA patients with renal papillary cell carcinoma to establish a risk signature of 15 IRGs. Similar IRG‐based prognostic signatures have been reported for gastric cancer,^29^ invasive ductal carcinoma^30^ and ovarian cancer^31^ as well. Based on these studies, our aim was to establish an immune‐related prognostic signature for CRC.

Here, we systematically analysed the immunogenomic landscape of CRC based on the gene expression profiles in TCGA and identified 476 differentially expressed IRGs between tumour samples relative to normal tissues including 18 survival‐associated IRGs. An IRG prognostic signature including *SLC10A2, FGF2, CCL28, NDRG1, ESM1, UCN, UTS2* and *TRDC* was constructed which showed moderate predictive ability for the overall survival of CRC patients in both the training and validation sets. Furthermore, this signature correlated with the tumour stage, invasion, lymph node metastasis and distant metastasis, and was identified as an independent prognostic indicator for CRC. This IRG signature may reflect the immune dysregulation in the tumour microenvironment and is a promising novel therapeutic target in addition to being an accurate prognostic biomarker for CRC.

## 2 MATERIALS AND METHODS

### 2.1 Data acquisition and IRG selection

RNA‐sequencing and clinical data of 568 CRC and 44 normal tissue samples were downloaded from TCGA database (https://portal.gdc.cancer.gov/)^32^ as 15 August 2019. A total of 2,498 IRGs (Table S1) associated with human cancers were identified using the Immunology Database and Analysis Portal (ImmPort) database (https://www.immport.org/home).^33^

### 2.2 Identification of differentially expressed IRGs

The limma R package^34^ was used to identify IRGs that were differentially expressed between the tumour and normal tissue samples, with false discovery rate (FDR) of < 0.05 and log2‐fold change > 1 as the cut‐off values. Gene Ontology (GO)^35^ and the Kyoto Encyclopedia of Genes and Genomes (KEGG) pathway analysis^36^ were conducted using the clusterProfiler R package^37^ to identify the functionally enriched genes and classify the gene clusters. FDR < 0.01 was considered statistically significant.

### 2.3 Survival‐associated IRG analysis

The survival‐associated IRGs were screened using data from patients surviving at least 90 days and with known M stage (pM), tumour stage (pStage), T stage (pT) and N stage (pN) [according to American Joint Committee on Cancer (AJCC)]. Accordingly, the data set of 453 patients was randomly assigned to the training (362 patients, 80% of all samples) and validation (91 patients, 20% of all samples) groups, and the survival‐related IRGs were identified by univariate Cox analysis with the survival R package (*P* < .05).

### 2.4 Construction and verification of the immune signature

Based on the results of the univariate analysis, the immune‐related prognostic signature was generated using a stepwise multivariate Cox proportional hazards model with the survival package in R language. Here, a multivariate Cox proportional hazards regression model was used to solve the problem of multiple factors affecting the survival time of patients. In brief, we considered all the 18 IRGs that are significantly related to prognosis by univariate Cox analysis as influencing factors. After bringing them into multivariate Cox proportional hazards model, significant IRGs will be retained during multiple computing. The weighted coefficients based on individual gene expression levels were used to calculate the risk score as follows:

$$\text{Risk score}=\sum \text{regression coefficient}\left({\text{gene}}_{i}\right)\times \text{expression value}\left({\text{gene}}_{i}\right).$$

The patients in the training group were then stratified into the low‐ and high‐risk groups according to median risk score values, and their survival was analysed using the Kaplan‐Meier method and the log‐rank test. The specificity and sensitivity of the risk score in predicting 3‐ and 5‐year survival was determined by ROC analysis using the survival ROC R package, and the areas under curve (AUC) were calculated. The immune signature was further validated using the entire TCGA (n = 453) and validation (n = 91) data sets, as well as the GSE38832 data set^38^ downloaded from the Gene Expression Omnibus (GEO) database.^39^ The latter included 115 CRC samples and the survival information in accordance with the GPL570 (Affymetrix Human Genome U133 Plus 2.0 Array). Each data set was stratified into the low‐ and high‐risk groups as described.

### 2.5 Association of the immune signature and clinicopathological features

The correlation between patient survival and clinical factors including age, gender, pM, pN, pT, pStage and risk scores was determined by univariate Cox analysis. Multivariate Cox regression analysis was then used to identify the independent prognostic factors for CRC. Prognostic nomograms were generated using the Cox regression coefficients with the rms R package, and the calibration plots to assess the performance of these nomograms were drawn using the regplot R package.

### 2.6 Bioinformatics analyses

The biological relevance of the immune signature was determined by gene set enrichment analysis (GSEA) using the GSVA R package. FDR < 0.001 and a relationship coefficient of > 0.3 were used as the cut‐offs for the included KEGG pathways. The association between the immune‐related genes was analysed by the Spearman correlation coefficient. The protein‐protein interaction network was based on the STRING database (https://string‐db.org/cgi/input.pl)^40^ and constructed using with an interaction confidence of 0.4 and 10 neighbouring nodes. The differentially expressed transcription factors (TFs) were identified from the Cistrome database (http://cistrome.org/),^41^ with FDR < 0.05 and log2‐fold change > 1 as the criteria. TF correlation assay was performed using *P* < .001 and correlation coefficient > 0.3 as the cut‐offs. The association between CRC biomarkers like *BRAF*,^42^ *NRAS* ^42^ and *PIK3CA*,^43^ and the immune prognostic signature was then determined. Finally, a competing endogenous RNA (ceRNA) regulatory network based on eight biomarkers was constructed using the TarBase (version 8)^44^ and LncBase (version 2)^45^ databases, and visualized using Cytoscape software (version 3.7.2).^46^

### 2.7 Clinical specimens

The expression of IRGs was clinically validated in 25 pairs of CRC and matched normal tissues surgically resected at the Second Affiliated Hospital of Zhejiang University School of Medicine from May 2018 to June 2018. These patients had received no preoperative chemotherapy, radiotherapy or targeted therapy. The tissue samples were collected within 30 minutes of surgical resection, cleaned and cryopreserved in liquid nitrogen. All patients provided informed consent, and the study was approved by The Human Research Ethics Committee of the hospital.

### 2.8 Real‐time quantitative polymerase chain reaction (RT‑qPCR)

Total RNA was extracted from the frozen tissue specimens using Trizol (#G3013, Servicebio) as per the manufacturer's instructions. The concentration and purity of the RNA were ascertained with a NanoDrop2000 spectrophotometer (ThermoFisher Scientific). Reverse transcription into complementary DNA (cDNA) was performed using a specific kit (#K1622, ThermoFisher Scientific) following the manufacturer's instructions. In brief, a total of 5 μg of total RNA (diluted to 12 μL using RNase‐free dH2O with 1 μL oligo(dT)_18_) were incubated at 65°C for 5 minutes and cooling on ice. Then, a 8 μL reaction mixture containing 4 μL of 5 × Reaction Buffer, 2 μL Of 10 mmol/L dNTP Mix, 1 μL of RiboLock RNAase inhibitor and 1 μL of RevertAi M‐MuLV reverse transcriptase was added in above RNA mixture (20 μL in total). The samples were finally incubated in a PCR thermocycler for 60 minutes at 42°C and 5 minutes at 70°C. RT‐qPCR was conducted in the StepOnePlus cycler (Applied Biosystems Inc) using FastStart Universal SYBR Green Master (#G3008, Servicebio). The PCR cycle parameters were as follows: pre‐denaturation at 95°C for 10 minutes, followed by 40 cycles at 95°C for 15 seconds and 60°C for 1 minute. Relative gene expression levels were measured using the comparative cycle threshold (ΔΔCt) method and normalized to GAPDH. The sequences and accession numbers for primers used in real‐time RT–PCR were shown in Table 1 (Servicebio). All samples were tested in triplicate.

**TABLE 1:** The information of primers used in real‐time RT–PCR

Gene	Sequences of primers	Accession No.	Tm (°C)	Amplicon (bp)
SLC10A2	FOR: AGGTGCCGAACGGTTGCTT	NM_000452.2	60°C	113
	REV: AGCGGGAAGGTGAATACGACA		60°C	
ESM1	FOR: CTTGCTACCGCACAGTCTCA	NM_001135604.2	60°C	124
	REV: GCCATGTCATGCTCTTTGCAG		60°C	
GAPDH	FOR: GGAAGCTTGTCATCAATGGAAATC	NM_002046	60°C	168
	REV: TGATGACCCTTTTGGCTCCC		60°C	

Abbreviations: FOR, forward; REV, reverse; Tm, Annealing temperatures.

### 2.9 Statistical analysis

All statistical analyses were conducted using R language (version 3.6.1). The Wilcoxon test was used to compare two independent non‐parametric samples, and the Kruskal‐Wallis test was used for multiple independent samples. The Kaplan‐Meier survival curves were compared with the log‐rank test. Independent prognostic factors related to survival were identified using the univariate and multivariate Cox proportional hazard regression analyses. The Spearman correlation coefficient was used to analyse the association among immune‐related genes. *P*‐value < .05 was considered statistically significant.

## 3 RESULTS

### 3.1 Construction of an immune‐gene expression signature in CRC

**FIGURE 1:**
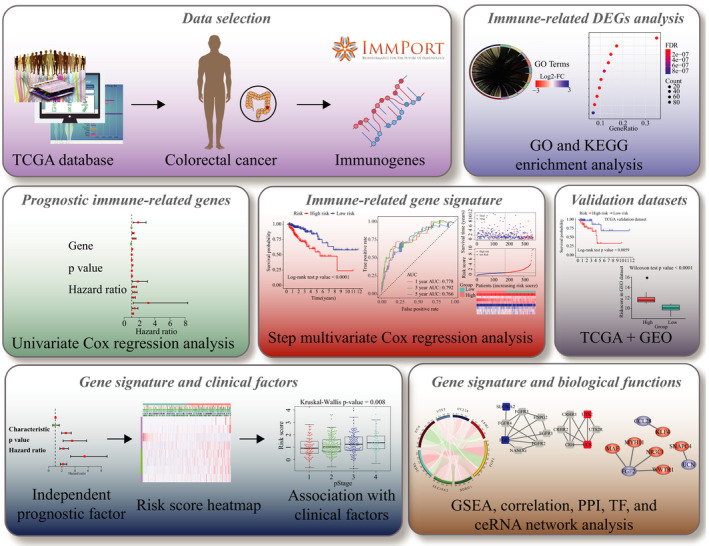
Flow chart for the development and verification of an immune‐related prognostic signature for CRC

**FIGURE 2:**
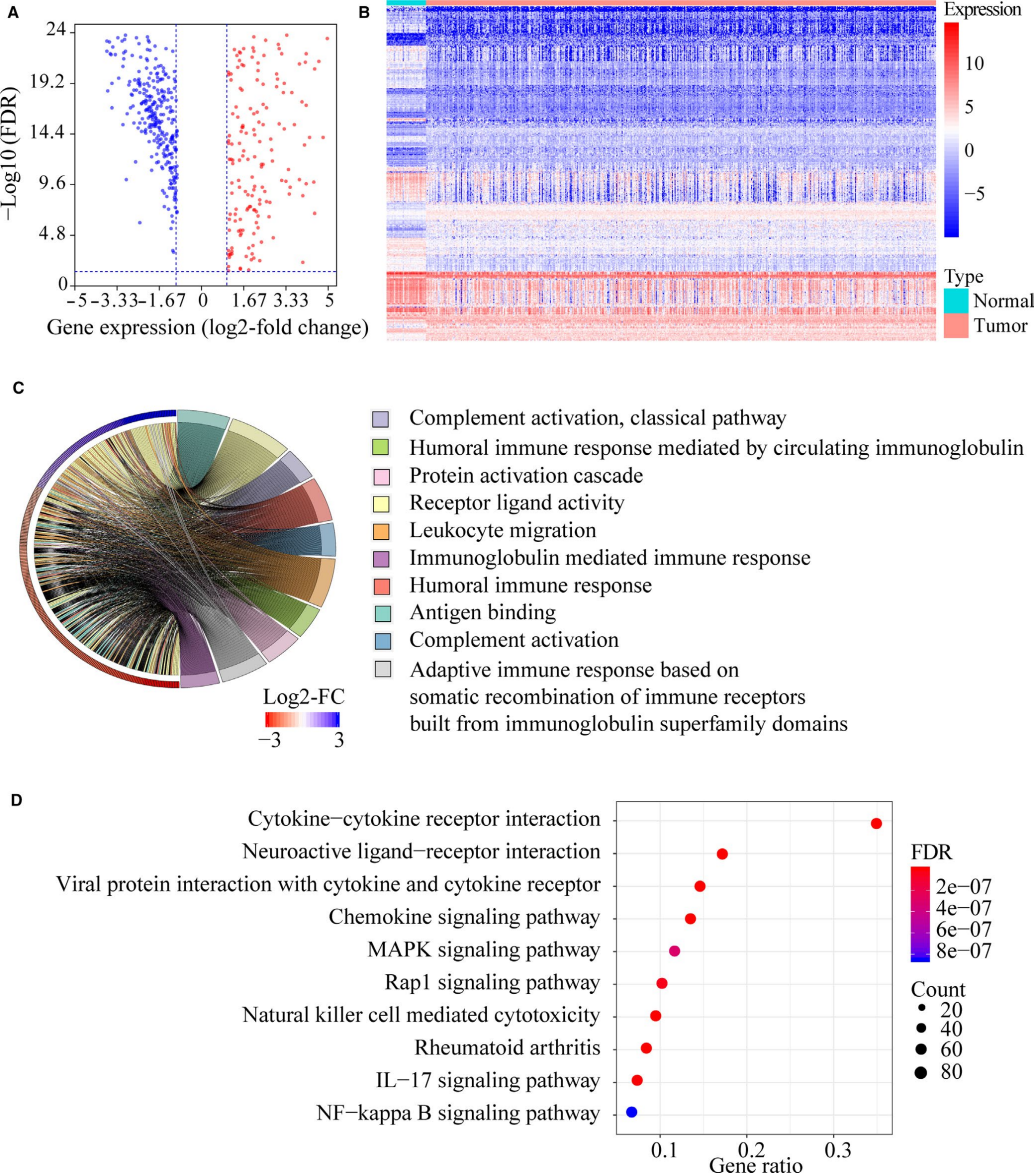
Screening of differentially expressed immune‐related genes (IRGs) in colorectal cancer (CRC). A, Volcano plot showing the differentially expressed IRGs in tumours vs normal tissue samples. Blue dots represent down‐regulated IRGs, and red dots represent up‐regulated IRGs. B, Gene expression heat map of differentially expressed IRGs in CRC. C, Results of the gene ontology (GO) term enrichment study. D, Results of the Kyoto Encyclopedia of Genes and Genomes (KEGG) pathway enrichment study

Given the crucial role of the immune microenvironment in cancer development, we explored the prognostic value of IRGs in CRC (Figure 1). Screening of TCGA and ImmPort databases revealed 476 IRGs that were differentially expressed in CRC compared to normal tissues, of which 177 were up‐regulated and 299 down‐regulated (Table S2; Figure 2A,B). The differentially expressed IRGs were significantly enriched in GO terms related to complement activation, humoural immune response, protein activation, immunoglobulin‐mediated immune response, antigen binding, phagocytosis, immunoglobulin complex formation and cytokine activity (Figure 2C and Table S3), and in cytokine‐cytokine receptor interaction, IL‐17 signalling pathway, natural killer cell‐mediated cytotoxicity, chemokine signalling pathway, Rap1 signalling pathway and MAPK signalling pathway (Figure 2D and Table S4).

**FIGURE 3:**
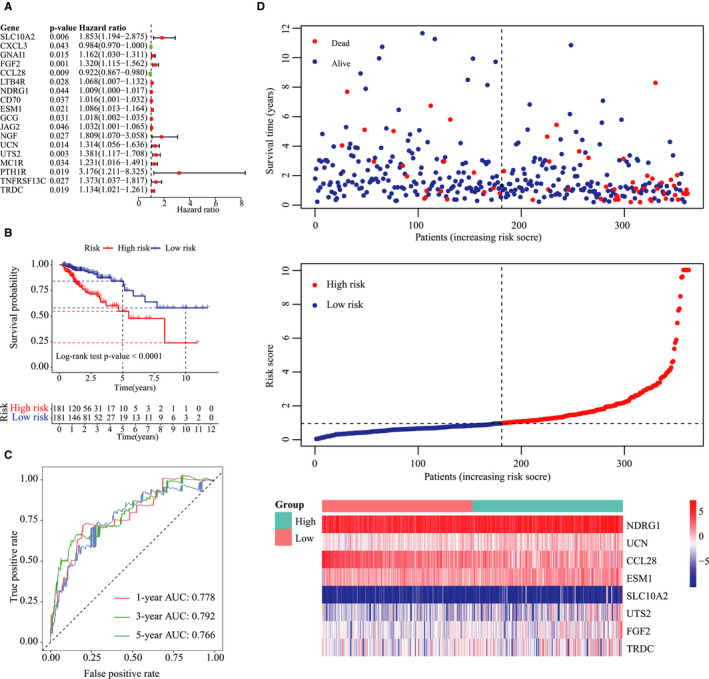
Construction of the immune‐related prognostic signature in CRC. A, Forest plot of immune‐related prognostic genes based on univariate Cox regression analysis. B, Kaplan‐Meier plots of the immune‐related signature showing worse survival in the high‐risk group compared to the low‐risk group (log‐rank *P*‐value < .0001). C, Time‐dependent (ROC) curve of the immune‐related signature for 1‐, 3‐ and 5‐year overall survival. D, Distribution of the survival status, risk score and gene expression data of CRC patients in the training group

Eighteen survival‐associated IRGs were identified in the training group by univariate Cox analysis (Figure 3A), of which *SLC10A2*, *FGF2*, *CCL28*, *NDRG1*, *ESM1*, *UCN*, *UTS2* and *TRDC* were further screened by the stepwise multivariate Cox proportional hazards model (Table 2). An eight‐gene immune signature was constructed using the independent regression coefficients of each gene, and the risk score was calculated as (0.639 * level of *SLC10A2*) + (0.387 * level of *FGF2*) + (−0.094 * level of *CCL28*) + (0.012 * level of *NDRG1*) + (0.124 * level of *ESM1*) + (0.378 * level of *UCN*) + (0.254 * level of *UTS2*) + (0.129 * level of *TRDC*). The risk scores were calculated for each patient in the training group from 0.02 and 24.80, and the patients were assigned to the high‐risk or low‐risk group based on the median risk score of 0.95. As shown in Figure 3B, patients with high‐risk scores had significantly worse survival outcome than those with low‐risk scores (log‐rank test, *P* < .0001). Furthermore, the AUC of the risk score for 3‐year and 5‐year OS were 79.2% and 76.6%, respectively (Figure 3C). The survival status, risk scores and gene expression data of CRC patients in the training group are illustrated in Figure 3D.

**TABLE 2:** The results of stepwise multivariate Cox proportional hazards model

Gene symbol	Coef	HR	HR.95L	HR.95H	*P*‐value	Regulation
SLC10A2	0.638812	1.89423	1.202826	2.983063	.005834	Down
FGF2	0.386611	1.471984	1.234656	1.754931	1.63E‐05	Down
CCL28	−0.09426	0.910051	0.851113	0.973069	.005796	Down
NDRG1	0.011524	1.011591	1.003183	1.020069	.006806	Down
ESM1	0.124352	1.132414	1.057736	1.212365	.000354	Up
UCN	0.377663	1.458871	1.157773	1.838275	.001364	Up
UTS2	0.254362	1.289639	1.028137	1.617652	.02781	Up
TRDC	0.129341	1.138078	1.015503	1.275449	.026111	Down

Note

Coef represents regression coefficient of each gene.

Abbreviations: HR, hazard ratio; HR.95H, high 95% confidence interval of HR; HR.95L, low 95% confidence interval of HR.

### 3.2 Validation of the immune‐related signature

**FIGURE 4:**
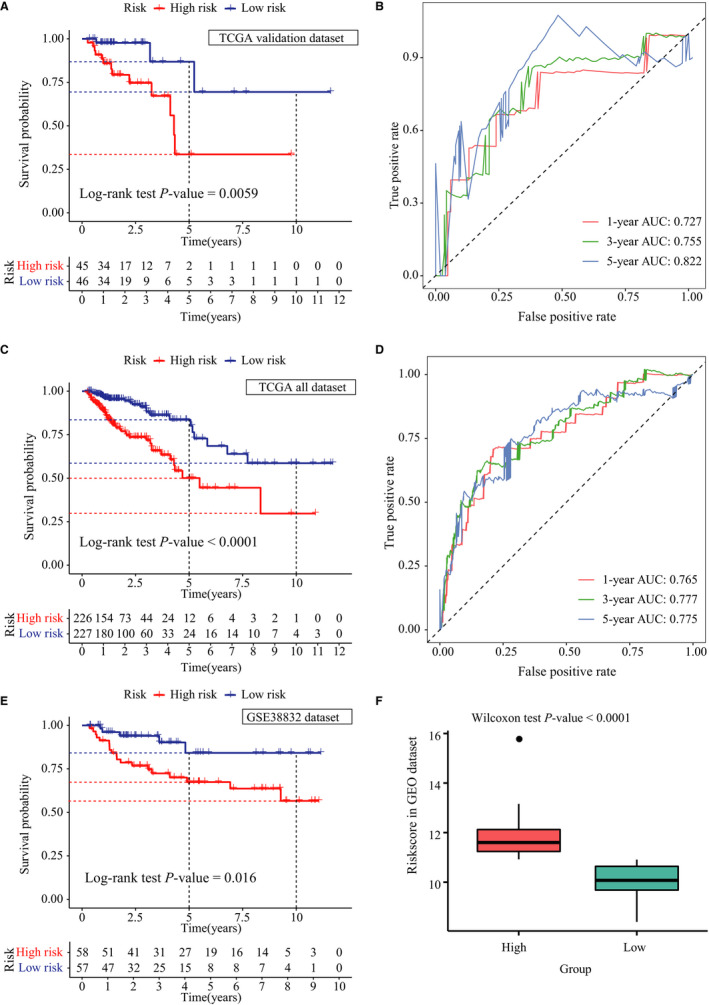
Verification of the immune‐related signature in three independent cohorts. A, Kaplan‐Meier plots of the immune‐related signature in the validation group. B, Time‐dependent receiver operating characteristics (ROC) curve of the immune‐related signature in the validation group. C, Kaplan‐Meier plots of the immune‐related signature in The Cancer Genome Atlas (TCGA) data set. D, Time‐dependent ROC curve of the immune‐related signature in TCGA data set. E, Kaplan‐Meier plots of the immune‐related signature in the GSE38832 database. F, Risk score distribution in low‐ and high‐risk groups

To validate the immune‐related signature, its prognostic accuracy was further assessed in three independent cohorts, including the validation group, TCGA data set and the GSE38832 data set. The OS was significantly longer for patients in the low‐risk vs the high‐risk group in the validation cohort (n = 91; log‐rank test, *P* = .006, Figure 4A), and the predicted 3‐year and 5‐year OS was 75.5% and 82.2%, respectively (Figure 4B). The TCGA data set (n = 453) also validated the prognostic accuracy of the immune‐related signature (log‐rank test, *P* < .0001, Figure 4C), with respective AUCs of 77.7% and 77.5% for 3‐year and 5‐year OS. Thus, the 8‐IRG immune signature predicted OS of CRC patients with superior accuracy (Figure 4D). Consistent with this, significantly longer OS was also seen for patients in the low‐risk group of the GSE38832 data set (n = 115) compared to those in the high‐risk group (log‐rank test, *P* = .016, Figure 4E). Furthermore, the risk scores were significantly different in both groups (Wilcoxon test, *P* < .0001, Figure 4F).

The expression level of the IRGs was validated in 25 matched tissue pairs by qRT‐PCR *ESM1* and *SLC10A2* showed the maximum differential expression between CRC and normal tissues, and consistent with the results of the bioinformatics analysis, *ESM1* was significantly elevated and *SLC10A2* was significantly down‐regulated (*P* < .05) in the tumours (Figure S1A,B).

### 3.3 The IRG signature confers additional prognostic power for CRC patients

**FIGURE 5:**
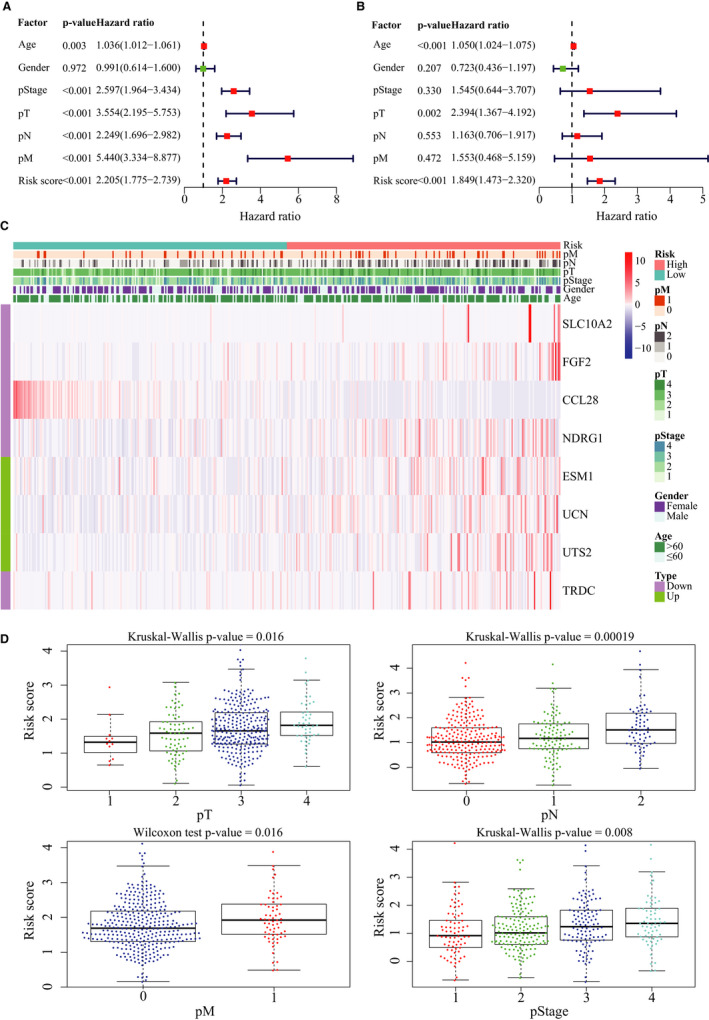
Association of the immune‐related signature with clinicopathological characteristics. A, Forest plot of risk scores and other clinical factors based on a univariable Cox regression analysis. B, Forest plot of risk scores and other clinical factors based on a multivariate Cox regression analysis. C, Expression profiles of the eight immune‐related genes. D, Box plots showing risk score distribution of different clinical factors

**FIGURE 6:**
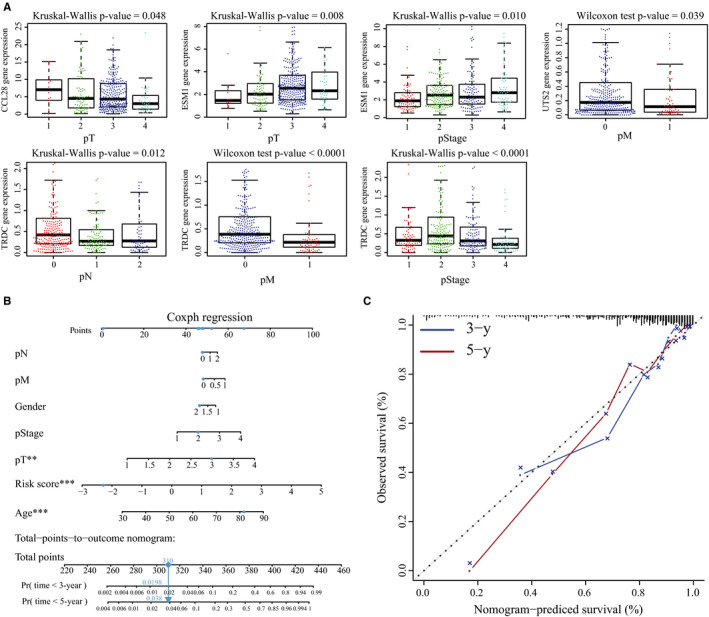
Expression of immune‐related genes (IRGs) associated with clinicopathological features, and construction of a nomogram for survival assessment. A, Associations between the different IRGs and clinicopathological features. B, Nomogram integrating the immune‐related signature to clinicopathological characteristics. C, Plots displaying the calibration of each model comparing predicted and actual 3‐ and 5‐year overall survival. The graph relative to the 45° line showing the model relative to perfect prediction

As shown in Figure 5A, clinical factors including age, pStage, pT, pN, pM and the immune risk score were closely associated with patient survival. Multivariate Cox regression analysis further showed that the IRG signature is an independent prognostic indicator for OS (*P* < .001, Figure 5B and Table 3). Expression profiles of the eight IRGs are shown in Figure 5C, and the risk score increased with advanced tumour parameters (Figure 5D). Furthermore, the expression level of *CCL28* was significantly associated with pT (*P* = .048), that of *ESM1* with pT (*P* = .008) and pStage (*P* = .01), *UTS2* with pM (*P* = .039), and *TRDC* with pN (*P* = .012), pM (*P* < .0001) and pStage (*P* < .0001, Figure 6A). We next created a nomogram that integrated clinicopathological characteristics with the IRG signature as a quantitative tool for predicting OS of CRC patients (Figure 6B). As shown in the calibration plots in Figure 6C, the nomogram performed with moderate accuracy compared to an ideal model.

**TABLE 3:** The univariate and multivariate Cox analysis for risk signature and clinical factors

Factors	Univariate Cox analysis		Multivariate Cox analysis	
	Hazard ratio (95% CI)	*P*‐value	Hazard ratio (95% CI)	*P*‐value
Age	1.036 (1.012‐1.061)	.003	1.050 (1.024‐1.075)	<.0001
Gender	0.991 (0.614‐1.600)	.972	0.723 (0.436‐1.197)	.207
pStage	2.597 (1.964‐3.434)	<.0001	1.545 (0.644‐3.707)	.330
pT	3.554 (2.195‐5.753)	<.0001	2.394 (1.367‐4.192)	.002
pN	2.249 (1.696‐2.982)	<.0001	1.163 (0.706‐1.917)	.553
pM	5.440 (3.334‐8.877)	<.0001	1.553 (0.468‐5.159)	.472
Risk score	2.205 (1.775‐2.739)	<.0001	1.849 (1.473‐2.320)	<.0001

Abbreviations: 95% CI, 95% confidence interval; pM: pathological metastasis stage; pN, pathological lymph node stage; pT, pathological tumour stage.

### 3.4 Biological revenant and regulatory network of the IRG signature in CRC

The potential association between the IRG signature and biological functions was assessed by GSEA using samples from TCGA data set. We found 28 KEGG pathways that were significantly correlated to either the low‐ or high‐risk CRC patient group (Figure S2A and Table S5). Pathways modulating cancer‐related functions such as focal adhesions, the actin cytoskeleton, ECM‐receptor interactions and endocytosis were positively correlated with the risk scores, while cytosolic DNA sensing, linoleic acid metabolism, arachidonic acid metabolism and ether lipid metabolism pathways were negatively correlated.

**FIGURE 7:**
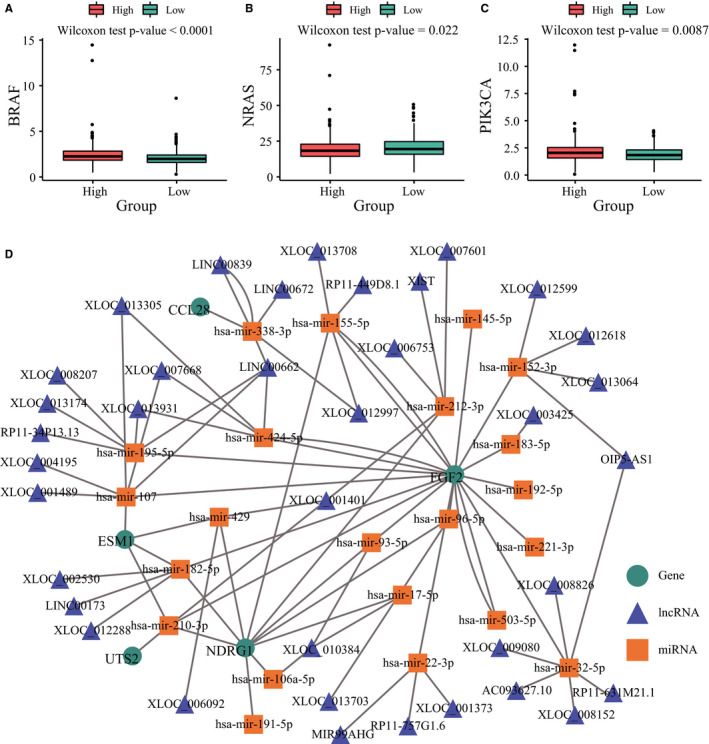
Correlation of immune‐related genes with biomarkers and construction of a competing endogenous RNA (ceRNA) network for colorectal cancer. A, *BRAF* gene expression in risk groups. B, *NRAS* gene expression in risk groups. C, *PIK3CA* gene expression in risk groups. D, The regulatory network of ceRNAs based on immune‐related genes

The potential correlation between the IRGs and other cancer‐related genes was also evaluated. As shown in Figure S2B, there was a significant negative correlation between *UCN* and *FGF2* (Spearman's correlation coefficient = −0.31), while the IRGs were weakly correlated with other genes. The interactions of the proteins encoded by these genes were next analysed (Figure S2C), and the Cistrome database further showed that *UCN*, *FGF2* and *CCL28* were regulated by transcription factors (Figure S2D). Finally, the expression of several CRC biomarkers was assessed in the tumours from the low‐ and high‐risk groups. *BRAF*, *NRAS* and *PIK3CA* were differentially expressed, indicating that the IRG signature is closely related to CRC progression (Figure 7A–C). Based on the above, a complex ceRNA network regulating IRGs was constructed using the TarBase and LncBase databases (Figure 7D).

## 4 DISCUSSION

The tumour microenvironment includes immune cells that are key players in tumour progression,^47^ and variously modulate inflammation^48^ and metastasis.^49^ Thus, both immunology‐cancer interface and the microenvironment have a major impact on the diagnosis and treatment of cancers, including CRC.^50^ Resistance to CRC immunotherapy is the result of poorly antigenic tumour cells displaying PD‐1 and PD‐L1.^14^ We systematically evaluated the immunogenomic landscape of CRC tumours based on gene expression profiles in TCGA database and constructed an immune‐related prognostic signature including *SLC10A2, FGF2, CCL28, NDRG1, ESM1, UCN, UTS2* and *TRDC*. The AUC values for 3‐year and 5‐year OS of this prognostic signature were 79.2% and 76.6%, respectively, indicating moderate predictive accuracy. Furthermore, the signature correlated with tumour stage, invasion, lymph node metastasis and distant metastasis, and is an independent predictor for the OS of CRC patients. This IRG signature likely reflects immune dysregulation in the tumour microenvironment and is a novel prognostic biomarker of CRC.

High throughput molecular analyses, gene expression databases and bioinformatic tools have enabled systematic profiling of immune signatures in cancers. For example, Yang et al^29^ built a prognostic model for gastric cancer consisting of immune‐related genes including *TNFRSF18, PBK, MICB, ITGA6, TLR5, PNMA1, LBP, CXCR4, C6* and *NRP1*, which accurately distinguished between patients with poor and satisfactory OS. Similarly, Bao et al identified an independent prognostic signature for invasive ductal carcinoma^30^ that consisted of *FLT3LG, SPIB, KLRB1, BATF, IGHA1, TIMM8A* and *QRSL1*. In this study, we identified an immune‐related signature comprising *SLC10A2*, *FGF2*, *CCL28*, *NDRG1*, *ESM1*, *UCN*, *UTS2* and *TRDC* for CRC. N‐myc downstream‐regulated gene 1 (*NDRG1*) was reported that it can play a key regulatory role in signalling pathways related to tumour progression, especially in tumour metastasis. Mi et al^51^ determined that *NDRG1* inhibits epithelial‐mesenchymal transition (EMT), migration and invasion by interacting and promoting caveolin‐1 ubiquitination in human CRC cells. Another study also proved that knockdown of *NDRG1* can promote EMT progress of CRC via NF‐κB signalling.^52^ *ESM1* that can be secreted into saliva, blood and urine is considered a candidate biomarker for CRC.^53^ The ceRNA‐ and TF‐mediated regulatory networks further identified *FGF2*, *CCL28* and *UCN* in CRC progression. *FGF2* is associated with increased CRC responsiveness to irinotecan^54^ and is also a member of a gene hub associated with cetuximab insensitivity in CRC.^55^ In addition, *CCL28* was previously identified as part of a prognostic signature that accurately predicted the OS of patients with CRC.^56^ Sun et al^57^ also identified CCL28 as a critical prognostic factor for CRC using GEO gene expression data and the rank aggregation method.

In human cancer studies, none of the single biomarkers can be used to detect cancers achieved the required specificity and sensitivity.^58^ When analysing one or two typical biomarkers, conflicting results are often obtained, leading to incorrect cancer diagnosis and unsuccessful treatment. Because it is known that several pathways and biological processes in tumour cells have changed, the concept of ‘single marker’ in cancer is incorrect.^59^ Some studies have reported extensive combinations of serum biomarkers in various cancers. The combination of serum biomarkers with nucleic acids including free DNA, mRNA, microRNA and circulating tumour DNA (ctDNA) is becoming a diagnostic tool for malignant tumours.^60^ In breast cancer, several recurrence prediction models including multi‐gene panels, such as Oncotype DX, EndoPredict, BCI and Curebest 95GC, were established.^61^ Therefore, above results may give us a hint: the combination of several biomarkers from different biological pathways can lead to better understanding of cancer progression and prognostic significance in solid cancers. However, the toxic side effects and other adverse reactions of multiple target genes therapy are uncertain, thus it is worth our serious considerations.

The prognostic IRGs in our study were enriched in 28 KEGG pathways, including cancer‐related pathways such as focal adhesions, the actin cytoskeleton, ECM‐receptor interactions and endocytosis. The actin cytoskeleton of cytotoxic lymphocytes is a major mediator of immune synapse formation and maturation,^62^ cell migration and immune surveillance.^63^ Focal adhesion is an essential step in cell migration, and the extracellular matrix (ECM) and its secreted cytokines play key roles in the immune escape of human tumours.^64^ The activation of several metabolism‐related pathways such as cytosolic DNA sensing, linoleic acid metabolism, arachidonic acid metabolism and ether lipid metabolism negatively correlated with the CRC risk scores. Thus, the IRG gene signature is biologically significant in CRC.

Our study has some limitations that should be addressed in future studies. First, transcriptomic analysis cannot reflect the molecular mechanisms underlying the immunobiology of CRC, which may be better elucidated by proteomics and/or metabolomics. Second, the robustness of our IRG signature must be verified in a large prospective clinical study. Finally, our findings have to be validated by in vitro and in vivo functional assays to further our understanding of the biological role of this IRG signature in CRC.

In conclusion, we identified and validated a novel immune‐related prognostic signature for patients with CRC, which likely reflects the immune dysregulation in the tumour microenvironment and is a potential prognostic biomarker and therapeutic target.

## CONFLICT OF INTEREST

The authors declare no conflicts of interest.

## AUTHOR CONTRIBUTION

**Jun Wang:** Conceptualization (lead); Formal analysis (lead); Writing‐original draft (lead); Writing‐review & editing (equal). **Shaojun Yu:** Methodology (lead); Writing‐review & editing (equal). **Guofeng Chen:** Conceptualization (supporting); Writing‐original draft (supporting). **Muxing Kang:** Conceptualization (supporting); Writing‐original draft (supporting). **Xiaoli Jin:** Methodology (supporting); Writing‐review & editing (equal). **Yi Huang:** Formal analysis (supporting); Methodology (supporting). **Lele Lin:** Formal analysis (supporting); Methodology (supporting). **Dan Wu:** Conceptualization (supporting); Writing‐original draft (supporting). **Lie Wang:** Writing‐review & editing (equal). **Jian Chen:** Writing‐review & editing (equal).

## Supporting information

Fig S1

Fig S2

Table S1

Table S2

Table S3

Table S4

Table S5

Supplementary Material

## Data Availability

All data generated or analysed during this study are included in this published article and its supplementary information files.
