# Supplementary figures and images for: A novel prognostic signature of immune‐related genes for patients with colorectal cancer
Source: J Cell Mol Med. 2020 Jun 21;24(15):8491–504. doi: 10.1111/jcmm.15443 (PMC7412433; doi:10.1111/jcmm.15443)

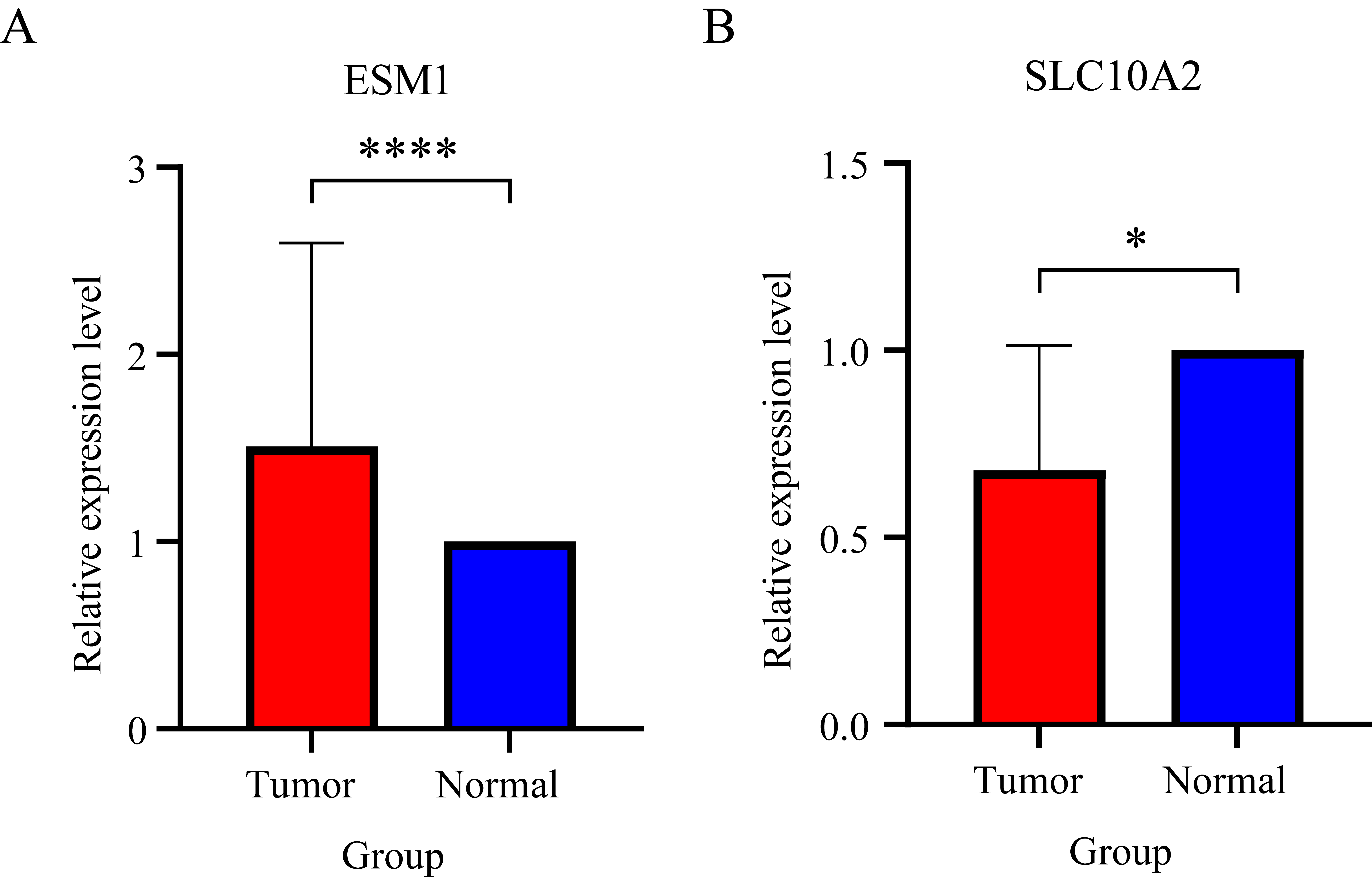

Supplement: Supplementary file 1 — Fig S1 [file JCMM-24-8491-s001.tif]

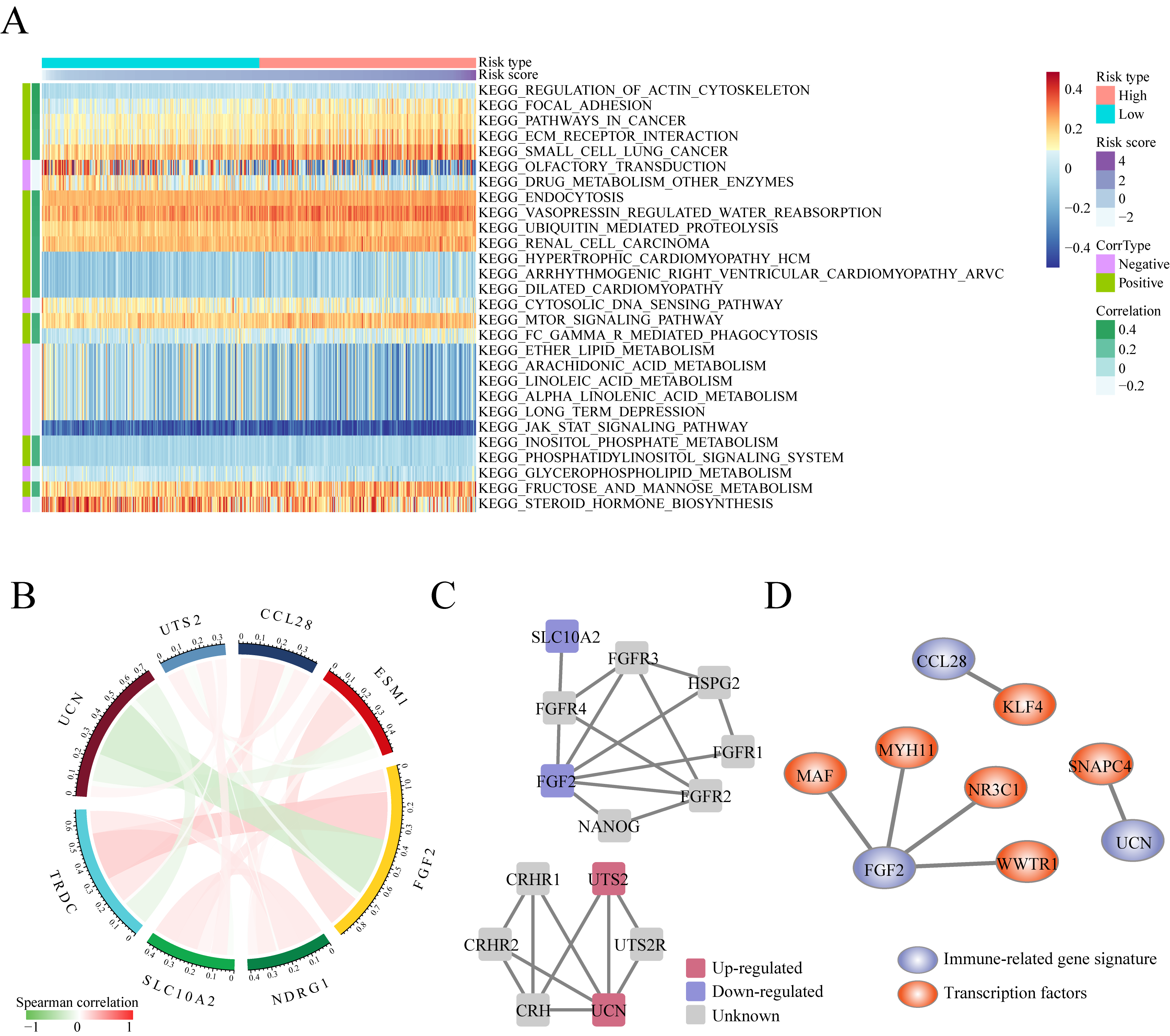

Supplement: Supplementary file 2 — Fig S2 [file JCMM-24-8491-s002.tif]
