# Supplementary material for: A novel prognostic signature of immune‐related genes for patients with colorectal cancer
Source: J Cell Mol Med. 2020 Jun 21;24(15):8491–504. doi: 10.1111/jcmm.15443 (PMC7412433; doi:10.1111/jcmm.15443)
Supplement: Supplementary file 8 — Supplementary Material [file JCMM-24-8491-s008.docx]

**SUPPLEMENTARY INFORMATION**

**SUPPLEMENTARY TABLE LEGENDS**

**Supplementary Table 1.** Immune-related gene (IRG) data from the ImmPort database.

**Supplementary Table 2.** Immune-related genes (IRGs) differentially expressed in colorectal cancer (CRC).

**Supplementary Table 3.** Gene ontology (GO) term enrichment analysis of differentially expressed immune-related genes (IRGs).

**Supplementary Table 4.** The Kyoto Encyclopedia of Genes and Genomes (KEGG) pathway enriched with immune-related genes (IRGs) differentially expressed in CRC.

**Supplementary Table 5**. Gene set enrichment analysis (GSEA).

**SUPPLEMENTARY FIGURE LEGENDS**

**Supplementary Figure 1. Validation of differentially expressed immune-related genes (IRGs) by RT‑qPCR.** (A) Comparative ESM1 mRNA levels in colorectal cancer (CRC) and normal tissues. (B) Comparative SLC10A2 mRNA levels in CRC and normal tissues; * p < 0.05, ** p < 0.01, *** p < 0.001, and **** p < 0.0001.

**Supplementary** **Figure 2. Association of the immune‐related signature with biological functions, and construction of a regulatory network.** (A) Relationship of the immune-related signature to the Kyoto Encyclopedia of Genes and Genomes (KEGG) pathways. (B) Correlation of the eight IRGs. (C) The protein-protein interaction network among immune-related genes (IRGs). (D) The regulatory network based on transcription factors and IRGs.
